# Literature in Plague Time

**Published:** 1889-02-16

**Authors:** 


*(To be continued.)*

### LITERATURE IN PLAGUE TIME.

In September, 1563, the Plague was raging in London. Queen Elizabeth was on the throne, but had not yet been able to persuade her otherwise loyal and obedient citizens that it was in their own interest that she commanded that no more building should go on within the City precincts, and that the crowded promiscuity in which the middle classes then lived was dangerous to health. The people continued to dwell in their narrow streets, where the gutter was the common sewer; the richest of them "heaped up together, and in a sort smothered with many children and servants," as the Queen's proclamation of 1602 phrased it, being little better off, from a sanitary point of view, than their poorest neighbours. And so the Plague broke out, not for the first nor the last time in those golden days when good Queen Bess was reigning.

These recurring epidemics had the effect of rendering one book so popular that it went through almost as many editions as a popular novel of our time. William Bullein's "Dialogue Against the Fever Pestilence"* first appeared in 1564 and speedily became popular. It had several claims on the attention of readers. It contained prescriptions for several compounds which were supposed to be specifics for the prevention or cure of the epidemic; interspersed with these were "merry tales" of the rather dreary mediæval kind, and solemn exhortations likely to fall on ready ears when death was taking toll on every side; lastly, there were sarcastic estimates of one or two popular characters—a species of literary fare that never palls on certain appetites. To us, who look at the book through the telescope of time, its chief interest lies in the picture it gives of life, thought, and custom in these bygone days.

As was customary in the old romances, the characters are types rather than individuals, as, indeed, is implied in their names: Civis, Uxor, Medicus, Avarus, Mendax, Theologus, and the rest. The miser, however, and the doctor—who is generally addressed, *more teutonico*, as "Maister doctor Tocrub"—seem to have been real personages, known to the author and not beloved by him. "Tocrub," it is generally supposed, is an anagram of Burcot, the name of a physician and alchemist, learned in the properties of metals and minerals, who had achieved some fame at the time, and is mentioned in several State papers, as well as in chronicles and works of imagination.

* "A Dialogue Against the Fever Pestilence." By William Bullein. From the edition of 1578, collated with the earlier editions of 1564 and 1573. Edited by Mark W. Bullein and A. H. Bullein. London: Published for the Early English Text Society by N. Trübner and Co., 57 and 59, Ludgate Hill. Price Ten Shillings.

The dialogue begins presumably at the door of a citizen's house. The attention of the occupant and his wife is attracted by a beggar, who repeats the Lord's Prayer as he goes along the street, much as our modern mendicants drone out Sankey's hymns. He is not a pious beggar, however, for he admits, with a frankness which we fear would be sought in vain in real life, that though he can say his pater-noster both in Latin and English, and the *De Profundis* too, he does not "ken nene of them bethe what they meane." But his parrot-prayer provokes a comment from Civis, the citizen, which shows that the *tutoiement*, now kept for the language of poetry and devotion in England, was formerly here, as elsewhere, that of familiarity. On hearing Mendicus drawl out, "Our Father which art in heaven, hallowed be Your name; Your kyngdom come; Your will be done," and so on, Civis remarks: "Methink I do hear a good mannerly beggar at the door, and well brought up. How reverently he says his Pater-noster; he 'thou's' not God, but 'you's' Him." Fashions change in all things with the ages, and to our ears the "you" in prayer jars as much as do the Parisian women in "Notre Dame," who civilly speak of "Madame la Vierge Marie."

